# Metal ions weaken the hydrophobicity and antibiotic resistance of *Bacillus subtilis* NCIB 3610 biofilms

**DOI:** 10.1038/s41522-019-0111-8

**Published:** 2020-01-03

**Authors:** Carolina Falcón García, Martin Kretschmer, Carlos N. Lozano-Andrade, Markus Schönleitner, Anna Dragoŝ, Ákos T. Kovács, Oliver Lieleg

**Affiliations:** 1 Department of Mechanical Engineering and Munich School of Bioengineering, Technical University of Munich, Boltzmannstraße 11, 85748 Garching, Germany; 2 Bacterial Interactions and Evolution Group, Department of Biotechnology and Biomedicine, Technical University of Denmark, Søltofts Plads 221, 2800 Kongens Lyngby, Denmark

**Keywords:** Biofilms, Antimicrobials, Bioremediation

## Abstract

Surface superhydrophobicity makes bacterial biofilms very difficult to fight, and it is a combination of their matrix composition and complex surface roughness which synergistically protects these biomaterials from wetting. Although trying to eradicate biofilms with aqueous (antibiotic) solutions is common practice, this can be a futile approach if the biofilms have superhydrophobic properties. To date, there are not many options available to reduce the liquid repellency of biofilms or to prevent this material property from developing. Here, we present a solution to this challenge. We demonstrate how the addition of metal ions such as copper and zinc during or after biofilm formation can render the surface of otherwise superhydrophobic *B. subtilis* NCIB 3610 biofilms completely wettable. As a result of this procedure, these smoother, hydrophilic biofilms are more susceptible to aqueous antibiotics solutions. Our strategy proposes a scalable and widely applicable step in a multi-faceted approach to eradicate biofilms.

## Introduction

Biofilm surface hydrophobicity has recently gained increasing attention.^1–8^ This is due to the improved protection bacteria experience within biofilms when a strong liquid repellency is present on the biofilm surface—and this has severe implications for medical and industrial processes. Specifically, *Bacillus subtilis* bacterium, a model organism for biofilm formation, has been reported to form biofilm surfaces with extremely strong hydrophobic (sometimes even omniphobic) properties.^1–8^ Those liquid-repellent biofilm surfaces can be further classified into rose- and lotus-like hydrophobic.^1^ The first category refers to a surface where liquid droplets stick when the surface is tilted; this is due to strong adhesion forces acting between the surface and the liquid droplet. In the second scenario, adhesive forces between the liquid and the surface are low, thus (e.g., water) droplets easily roll off once the surface is tilted. Both types of superhydrophobic behavior are widely found in nature, and their names are derived from the plant leaves where this behavior has been studied the most.^9–12^

Although they exhibit similar liquid repellency, plant leaves and bacterial biofilms are very different materials. The latter is a highly hydrated, viscoelastic slime comprising a mix of biopolymers into which the bacteria embed themselves. Depending on the bacterial strain, the macromolecular composition of the biofilm matrix can be very different. In fact, each bacterial strain can secrete a unique mixture of extracellular polymeric substances (or EPS). For instance, three major components have been identified to constitute the biofilm matrix of *B. subtilis* NCIB 3610: a hydrophobin protein (BslA); an exopolysaccharide produced by the *epsA-O* operon; and a fiber-forming protein (TasA). Each component is suggested to have one or more particular functions: the hydrophobin protein forms a coat on the biofilm surface, thus critically contributing to its water repellency.^4,6,13,14^ The exopolysaccharide, whose synthesis is required for the BslA proteins to localize to the biofilm matrix, bundles the cells and contributes—together with BslA—to the final roughness and height of *B. subtilis* biofilms.^4,15,16^ Finally, the fibers formed by TasA protein connect the cells within the matrix, thus rendering the structure of the biofilm more compact and stable.^15,17^ Hence, a combination of chemical and physical contributions, that is, hydrophobic molecules and surface roughness features (the latter of which are established by a complex combination of different biofilm matrix components), gives rise to the highly efficient wetting resistance of those biofilms.

Although vastly different in terms of biochemical composition, the detailed mode of superhydrophobicity of both, biofilms and plant leaves, is dictated by the topography of the material surface. Previously, we have shown that lotus- and rose-like biofilms exhibit highly complex but different surface features.^1^ We suggested that the two variants of biofilm superhydrophobicity can be described by similar physical models, which are already used to rationalize the wetting resistance of rose petals and lotus leaves: the impregnated Cassie and the Cassie–Baxter model, respectively. Both physical models describe complex surfaces with hierarchical roughness features (i.e., in the nano- and micro-scale). However, there is a key difference: whereas the liquid is in contact with the microstructures of rose-like surfaces, on lotus-like surfaces microscopic air pockets separate the liquid from the solid phase.^10,12,18–20^ Therefore, it is not surprising that lotus-like superhydrophobicity provides biofilms with a supreme physical protection mechanism against antimicrobials— especially when such agents are mostly available as aqueous solutions.^2,3,13,21^

To fight biofilms and to chemically inactivate the biofilm bacteria, it is crucial to efficiently access the bacteria within the biofilm matrix with antimicrobial agents. Studies aiming at disrupting the protective biofilm matrix mainly focus on the enzymatic degradation of EPS components or the disassembly of the matrix architecture by antibodies, microbial surfactants, or nucleic acid-binding proteins.^22,23^ Similarly, biological macromolecules (i.e., mucin glycoproteins or the alginate oligomer oligoG) have been proposed to promote the disassembly of *Pseudomonas aeruginosa* biofilms.^24,25^ However, such efforts are mostly strain specific and—when involving antibodies or purified biomolecules—expensive. This makes it difficult to implement them on large scales, that is, for industrial applications.

A simpler way to gain access to biofilm bacteria would be to weaken or remove the superhydrophobic properties of the biofilm surface. Previously, we introduced a strategy to modify the surface roughness of mature biofilms, which resulted in a reduced or even vanished water repellency. There, this was achieved by exposing the biofilm surface to highly concentrated salt or sugar solutions.^2^ The ensuing topographical changes of the biofilm surface entailed a more effective chemical inactivation of biofilm bacteria as well as a facilitated mechanical removal of the biofilm material. However, this approach requires very high concentrations of ions or carbohydrates to take effect, and the incubation times needed for achieving a weakening of the biofilm wetting resistance can be as high as 48 h. Also, high concentrations of salts or sugars can lead to unwanted side effects, such as accelerating corrosion on the biofilm-colonized material or serving as nutrients for other microorganisms.

Of course, there are also biofilm control strategies, which are applied before a biofilm has been established. Examples include the inhibition of EPS synthesis (by targeting signaling pathways) or preventing the colonization of surfaces by bacteria (by means of surface patterning and/or antimicrobial coatings).^22,26^ In this context, metal ions such as Cu^2+^ and Zn^2+^ have been widely explored as biofilm inhibitors and antimicrobial agents.^27–29^ For instance, antimicrobial metallic surfaces made of copper are suggested to induce lipid peroxidation in bacteria, thus causing impaired membrane function.^30^ Zinc oxide nanoparticles have been shown to inhibit bacterial growth and biofilm formation.^31^

Inspired by those findings, we here test the impact of (low concentrations) CuSO_4_ and ZnCl_2_ on the biofilm matrix of *B. subtilis* NCIB 3610, both at early and late stages of biofilm formation. Using a combination of microbiological and biophysical methods, we show that both metal ions cause an unspecific reduction of the expression of biofilm matrix-promoting genes. This is accompanied by changes in both the biofilm surface roughness and wetting behavior. As a result, biofilms grown in the presence of metal ions are more susceptible to treatment with aqueous antibiotic solutions. Interestingly, a similar effect is obtained when the metal ions are diffused through the substrate on which mature biofilms have been already grown. Based on our results, we propose that the use of low concentrations of metal ion solutions could be a low-risk, cost-effective, and widely applicable strategy for biofilm control—even in settings where such sturdy biomaterials have already been formed.

## Results and discussion

### Surface topography and wetting properties of *B. subtilis* NCIB 3610 biofilms are strongly altered when cultivated in the presence of metal ions

**Fig. 1:**
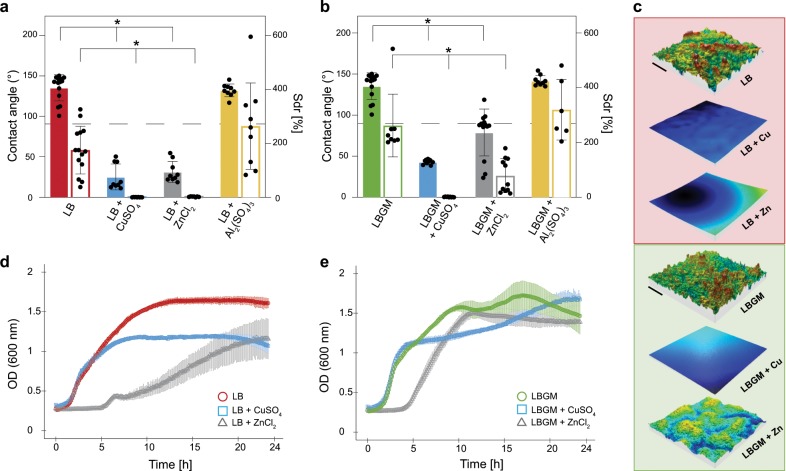
Effect of metal ions on *B. subtilis* NCIB 3610 biofilms surface wetting and topography, and impact on growth kinetics of planktonic bacteria. **a**, **b** Contact angle (solid bars) and Sdr (empty bars) values of biofilms cultivated on LB (**a**) and LBGM (**b**) agar with and without one of the following metal ions: CuSO_4_ (1.5 mM), ZnCl_2_ (0.5 mM), or Al_2_(SO_4_)_3_ (1.5 mM). The dashed lines separates hydrophilic (<90°) from hydrophobic (>90°) behavior. Asterisks denote statistically significant differences as assessed by a one-way ANOVA or Welch’s ANOVA using *p* = 0.05 (see Methods). **c** Profilometric images of biofilms grown at standard and metal ions enriched conditions, the scale bar denotes 0.2 mm in *x* and *y*; the *z* scale is max. 110 μm for LB samples (red background denoting rose-like wetting behavior in standard conditions) and 150 µm for LBGM samples (green background denoting lotus-like wetting behavior in standard conditions). **d**, **e** OD_600_ values are determined for planktonic bacteria grown in LB (**d**) and LBGM (**e**) liquid media over time, both at standard and metal ions enriched conditions. Error bars denote the standard deviation (s.d.), which was determined as follows: **a**, **b** from at least 6 individual data points, obtained from a minimum of 3 growth batches with a minimum of 2 biological replicates, and 1 technical replicate each (*B* ≥ 3, *N* ≥ 2, *n* = 1); **d**, **e** from at least 12 individual data points, obtained from a minimum of 3 growth batches with 1 biological replicate, and a minimum of 4 technical replicates each (*B* ≥ 3, *N* ≥ 1, *n* ≥ 4).

When *B. subtilis* NCIB 3610 bacterium is cultivated on Luria-Bertani (LB) medium at 30 °C for 24 h, biofilms with moderate surface roughness (Sdr ~175%) and rose-like wetting behavior are formed (Fig. 1a). In contrast, when the cultivation medium is enriched with glycerol and manganese sulfate (=LBGM medium^32^) biofilms with a higher surface complexity (Sdr ~260%) are obtained, and such biofilms show a lotus-like wetting behavior (Fig. 1b). The formation of *B. subtilis* biofilms with strongly liquid-repellent surfaces has been observed at different growth conditions and on different liquid and semi-solid agar substrates.^1–3,7^ However, here, only those instances, where the two types of superhydrophobic behavior can be observed, are selected; that is, biofilms are cultivated on LB agar to obtain rose-like behavior and on LBGM agar to obtain lotus-like behavior. It is observed that, regardless of the original biofilm characteristics, when the medium in the agar substrate is supplemented with ZnCl_2_ or CuSO_4_, the same bacterial cells almost always generate biofilms whose surface hydrophobicity is lost. At the same time, the roughness features on these biofilm surfaces are greatly reduced (Fig. 1a–c). Only for wild-type (WT) biofilms cultivated on LBGM medium, the presence of ZnCl_2_ during biofilm growth leads to colonies with more variable wetting behavior (Fig. 1b). Whereas colonies with hydrophilic surfaces are obtained in most cases, some colonies exhibit hydrophobic behavior. Still, also here, when compared to biofilms cultivated on standard LBGM agar, a significant reduction in the average surface hydrophobicity is achieved by the presence of ZnCl_2_. Interestingly, the opposite effect is observed when the same bacteria are cultivated on Al_2_(SO_4_)_3_-enriched agar; here, the biofilms show increased surface complexity and their hydrophobic character is maintained (Fig. 1a, b). The reason for testing this subset of metal ions is twofold. First, such metal ions are chosen that are likely to come in contact with bacteria in the environment/industrial settings, for example, when growing in pipes. Also, based on previously reported effects of different metal ions on the elastic modulus of biofilms,^33,34^ it would not be surprising that these ions might induce other physical effects as well. Indeed, not only “negative” (induced by copper and zinc) but also “positive” (induced by aluminum) effects can be achieved by incubating NCIB 3610 bacteria with metal ions.

Although the concentrations of ZnCl_2_ and CuSO_4_ used here (0.5 and 1.5 mM, respectively) are sufficiently high to affect the macroscopic properties of biofilms (Fig. 1c), at the same time, these low concentrations are not sufficient to completely inhibit bacterial replication (Fig. 1d, e). Indeed, planktonic growth of *B. subtilis* only shows slightly altered kinetics when copper or zinc are present in the liquid media. In the case of LB medium, growth is retarded in the presence of zinc, whereas in the presence of copper, we measure similar growth kinetics as for the control—albeit with lower final optical density (OD) values (Fig. 1d). To test the impact of these variations in OD_600_ on cell viability, colony-forming unit (CFU) tests are conducted. When liquid cultures of NCIB 3610 bacteria grown in the three different media (LB, LB + CuSO_4_, and LB + ZnCl_2_) are tested for CFUs, similar values are obtained for all three samples (Supplementary Fig. 1). Interestingly, bacteria grown in the presence of zinc show a slightly higher final CFU/mL average than the control, that is, the LB samples (Supplementary Fig. 1). Additionally, although LBGM is regarded as a biofilm-promoting medium,^32^ planktonic growth is assessed in this medium as well. This is done to create similar conditions during planktonic growth (regarding nutrients and metal ions exposure) as when biofilms are cultivated on LBGM agar. A similar trend in OD_600_ values as for LB is obtained for growth in LBGM medium. Here, zinc delays the entry into exponential growth of WT *B. subtilis*, whereas in the presence of copper, a similar curve is obtained as for the control. For all growth tests conducted in LBGM media, the final OD values are similar (Fig. 1e).

The metal ions used here for supplementation of the different media are essential for many cellular functions of the bacteria; nevertheless, in excess, they can also be lethal. An antimicrobial activity of copper and zinc ions has been widely reported with regard to protein binding, reactive oxygen species production, and membrane impairment.^27^ Together, the previous observations show that the presence of subtoxic levels of selected metal ions during the cultivation of *B. subtilis* NCIB 3610 bacteria on a semi-solid substrate has a strong impact on the final morphological and physical characteristics of the formed biofilms. Also, these findings suggest that exposing mature biofilms to such metal ions could entail significant changes in the biofilm wetting resistance—and such a phenomenon could be used as a biofilm control strategy.

### *Bacillus subtilis* NCIB 3610 growth in the presence of copper and zinc ions shows an unspecific under-expression of biofilm-promoting genes

**Fig. 2:**
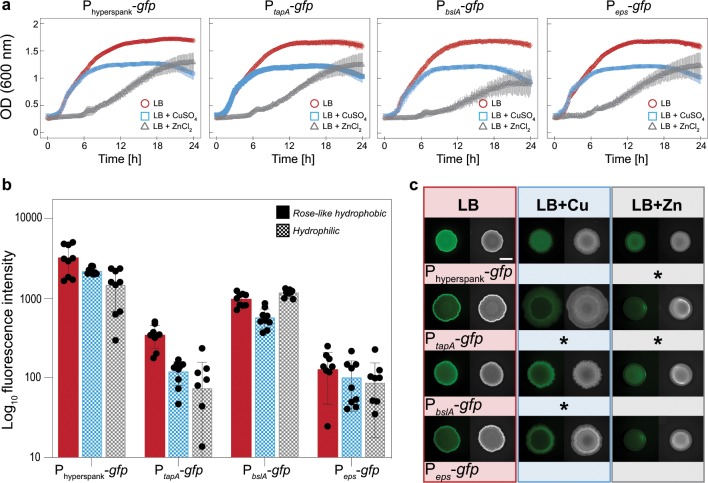
Effect of CuSO_4_ and ZnCl_2_ on planktonic growth and biofilm colony fluorescence using GFP-labeled *B. subtilis* NCIB 3610 strains and LB as a base growth medium. **a** OD_600_ values for planktonic bacteria grown in LB liquid medium over ~24 h, both in the presence and absence of CuSO_4_ or ZnCl_2_. **b** Relative fluorescence intensity values determined for biofilm colonies grown on LB agar with and without CuSO_4_ or ZnCl_2_ ions, respectively. Solid bars denote rose-like hydrophobicity, whereas checkered bars denote hydrophilic behavior. **c** Fluorescence and bright field images of the different biofilm colonies characterized in **b**; asterisks represent a significant reduction of fluorescence intensity values when compared to biofilms grown on standard LB agar, as assessed by a one-way ANOVA or Welch’s ANOVA with a *p* value of 0.05 (see Methods). The scale bar in **c** indicates 5 mm and is valid for all images. Throughout the figure, the colors indicate the type of media used for cultivation: red = LB; blue = LB + CuSO_4_; gray = LB + ZnCl_2_. Error bars denote the s.d., which was determined as follows: **a** from at least 6 individual data points, obtained from a minimum of 3 growth batches with 1 biological replicate, and a minimum of 2 technical replicates each (*B* ≥ 3, *N* ≥ 1, *n* ≥ 2); **b** from 9 individual data points, obtained form 3 growth batches with 3 biological replicates, and 1 technical replicate each (*B* = 3, *N* = 3, *n* = 1).

**Fig. 3:**
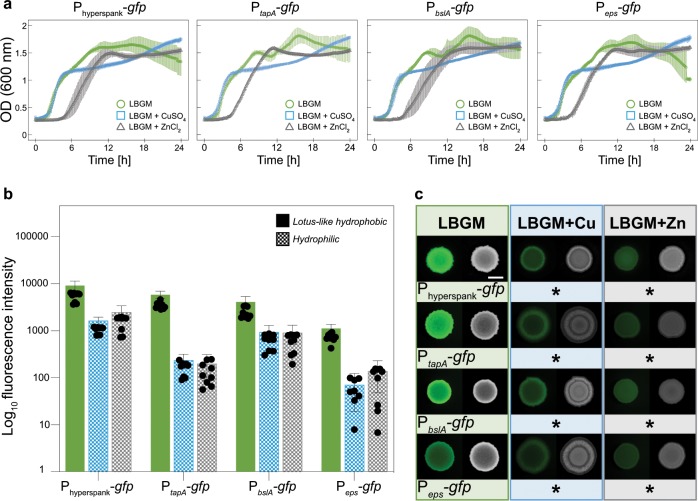
Effect of CuSO_4_ and ZnCl_2_ on planktonic growth and biofilm colony fluorescence using GFP-labeled *B. subtilis* NCIB 3610 strains and LBGM as a base growth medium. **a** OD_600_ values for planktonic bacteria grown in LBGM liquid medium over ~24 h, both in the presence and absence of CuSO_4_ or ZnCl_2_. **b** Relative fluorescence intensity values determined for biofilm colonies grown on LBGM agar with and without CuSO_4_ or ZnCl_2_ ions, respectively. Solid bars denote lotus-like hydrophobicity, whereas checkered bars denote hydrophilic behavior. **c** Fluorescence and bright field images of the different biofilm colonies characterized in **b**; asterisks represent a significant reduction of fluorescence intensity values when compared to biofilms grown on standard LBGM agar, as assessed by a one-way ANOVA or Welch’s ANOVA with a *p* value of 0.05 (see Methods). The scale bar in **c** indicates 5 mm and is valid for all images. Throughout the figure, the colors indicate the type of media used for cultivation: green = LBGM; blue = LBGM + CuSO_4_; gray = LBGM + ZnCl_2_. Error bars denote the s.d., which was determined as follows: **a** from at least 6 individual data points, obtained from a minimum of 3 growth batches with 1 biological replicate, and a minimum of 2 technical replicates each (*B* ≥ 3, *N* ≥ 1, *n* ≥ 2); **b** from 9 individual data points, obtained form 3 growth batches with 3 biological replicates, and 1 technical replicate each (*B* = 3, *N* = 3, *n* = 1).

It is generally accepted that it is a combination of complex roughness and chemistry dictated by all matrix components—and not a single matrix component alone—that allows biofilms to establish their characteristic liquid repellency. Therefore, our next aim is to test if the presence of metal ions during biofilm growth disturbs the formation of hydrophobic biofilms by affecting the expression of genes responsible for the production of biofilm matrix components. For this purpose, *B. subtilis* strains harboring the P_*eps*_-*gfp*, P_*bslA*_-*gfp*, and P_*tapA*_-*gfp* transcriptional reporters are used, which carry a promoter fusion to *gfp* gene. Additionally, a fourth reporter strain carrying a P_hyperspank_-*gfp* construct is used to assess the general expression in the cells. *B. subtilis* carrying the hyperspank promoter cloned before the reporter gene, but lacking the *lacI* repressor, is used to monitor general transcription in the bacterium. Transcription from this promoter has been previously detected to be constitutive in *B. subtilis* under planktonic and biofilm conditions.^35–37^ First, as described above for the WT strain, these reporter strains are incubated in liquid media to assess the challenge induced by the metal ions on the growth kinetics. It is observed that the metal ions alter the planktonic growth of all reporter strains in a similar way observed on the non-labeled WT (Figs 2a and 3a). Next, the different reporter strains are grown on LB- or LBGM agar enriched with copper (1.5 mM) or zinc (0.5 mM), and the surfaces of the biofilm colonies are imaged with fluorescence microscopy. From those images, fluorescence intensity values are calculated and compared to normal growth conditions, that is, to those obtained from biofilms grown on standard nutrient medium (LB or LBGM agar).

As illustrated in Fig. 2, initially hydrophobic rose-like biofilms (those grown on LB agar) show the highest average fluorescence intensity for the P_*bslA*_-*gfp* strain, followed by the P_*tapA*_-*gfp* and the P_*eps*_-*gfp* strains, respectively. When biofilms are grown in the presence of CuSO_4_, a significant reduction in fluorescence intensity is observed for biofilms formed by the P_*tapA*_-*gfp* and the P_*bslA*_-*gfp* strains, compared to those cultivated on standard LB agar (Fig. 2b, c). In contrast, when grown in the presence of ZnCl_2_, a significant reduction in fluorescence intensity is only observed for the P_*tapA*_-*gfp* strain, whereas the opposite trend is observed for the P_*bslA*_-*gfp* strain (Fig. 2b, c). The P_*eps*_-*gfp* strain was able to form biofilms with similar fluorescence intensity—independent of the media the strain was grown on (Fig. 2b, c).

When the reporter strains are grown on LBGM agar, where they generate lotus-like biofilms, similarly high average fluorescence intensity is measured for the P_*tapA*_-*gfp* and P_*bslA*_-*gfp* strains, whereas the P_*eps*_-*gfp* samples return the lowest values (Fig. 3b). However, when these biofilms are grown in the presence of copper or zinc, an overall under-expression of matrix-promoting genes is observed, and *tapA* and *eps* operons are most prominently affected (Fig. 3b, c). Importantly, the metal ions not only affect the expression of specific biofilm matrix components but also appear to have a general impact on fluorescent protein levels. For the control reporter strain (P_hyperspank_-*gfp*), similarly lower fluorescence intensity values are detected when grown on agar enriched with copper or zinc compared to standard LB or LBGM agar, respectively (Figs 2 and 3).

**Fig. 4:**
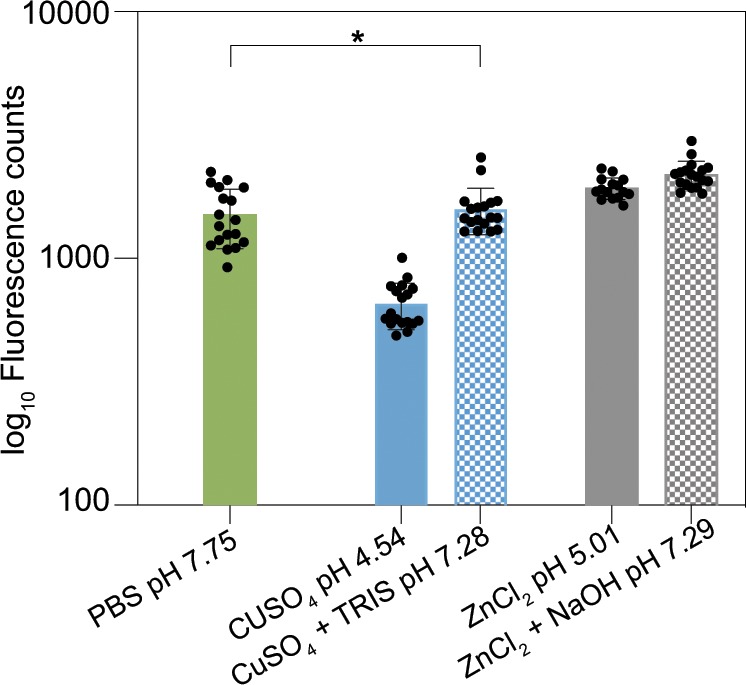
Effect of acidic pH induced by the addition of metal ion solutions on the fluorescence intensity of purified GFP. Solid bars represent unaltered solutions, whereas checkered bars represent neutralized solutions. No statistically significant difference was found between the solid green and the solid blue bars, as assessed by a one-way ANOVA with a *p* value of 0.05 (see Methods). Error bars denote the s.d. as determined from at least 15 individual data points, obtained from a minimum of 5 independent replicates with 3 technical replicates each (*N* ≥ 5, *n* = 3).

As an additional control, ensuring that the alteration in biofilm colony fluorescence we report here is not a result from a direct influence of the metal ions on the green fluorescent protein, the stability of this protein is tested in the presence of copper and zinc. Of course, the pH of solutions containing either CuSO_4_ or ZnCl_2_ is acidic, but it is indicated in the literature^38^ that green fluorescent protein (GFP) should be stable in a pH range from 6 to 10. When purified GFP mixed with CuSO_4_ or ZnCl_2_ solutions (at final concentrations of 1.5 and 0.5 mM, respectively) is analyzed both at acidic and neutral pH, the fluorescence intensity of the protein is not affected when the metal ion-containing solutions are neutralized with Tris buffer or NaOH (Fig. 4). Furthermore, the pH of the nutrient media used for bacterial cultivation is determined to be ~6.2 with 1.5 mM CuSO_4_ and ~6.7 with 0.5 mM ZnCl_2_, which is still in the range of stability reported for GFP.

Together, these results suggest that biofilm cultivation at metal ion-enriched conditions is possible but—in most cases—suffers from an unspecific under-expression of genes, and this includes diminishing the production of biofilm matrix components. Together with our assessment of biofilm wetting and topography discussed above, we conclude that this reduced availability of matrix components is responsible for a less-developed surface topography and thus a reduced wetting resistance of the biofilm colonies.

### Cultivation of *B. subtilis* NCIB 3610 biofilms in the presence of CuSO_4_ or ZnCl_2_ increases antibiotic efficiency

We have previously reported that biofilms with superhydrophobic surfaces are less susceptible to treatment with aqueous antibiotic solutions than biofilms with hydrophilic surfaces.^2^ Hydrophilic biofilm surfaces allow for full wetting and, subsequently, enable the entrance of an antibiotic molecule into the biofilm matrix. This is a necessary step for the biofilm bacteria to come in contact with the antibiotic so that it can take effect. On superhydrophobic biofilm surfaces, there is—in addition to a chemistry-based water repellency—a physical barrier provided by the roughness features. In the case of lotus-like superhydrophobicity, this complex topography entails the formation of a microscopic air cushion on the biofilm surface that largely prevents direct contact between the antibiotic and the biofilm bacteria. Thus, we next test if the two metal ions, Cu^2+^ and Zn^2+^, as a direct consequence of preventing the formation of superhydrophobic lotus-like biofilm surfaces, also increase the efficacy of selected antibiotics towards biofilm-embedded bacteria. As the microorganisms used here are non-pathogenic, our antibiotic treatment represents a conceptual model for the treatment of superhydrophobic biofilms formed by pathogenic bacteria. Due to the potential health risks associated with highly sturdy biofilms, it is important to study alternatives to eradicate them.

In brief, biofilms are cultivated on LBGM agar (in the presence and absence of metal ions), and the mature biofilms are scraped from their substrate and treated for 1 h with antibiotic aqueous solutions. The following antibiotics are used: piperacillin/tazobactam (8:1) (0.15 mg/mL), tetracycline (1.28 mg/mL), and kanamycin (1.28 mg/mL). The antibiotic concentrations correspond to ~150× of reported minimum inhibitory concentration (MIC_90_) values for *Bacilli*. Then, CFUs are determined to assess the number of viable cells after the antibiotic treatment (see Methods). In parallel, control samples are treated and assessed in the same way as stated before, but incubated in water instead of an antibiotic solution. The previous method allows us to determine a percentage of efficiency of the antibiotic treatment on the biofilm cells (see Methods).

**Fig. 5:**
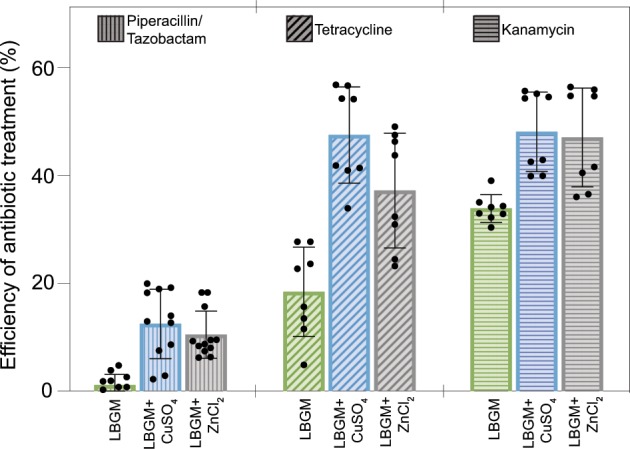
Effect of antibiotic treatments with aqueous solutions on *B. subtilis* NCIB 3610 biofilms that were cultivated in the presence and absence of metal ions. Efficiencies (in percentage values) are determined after a treatment of 1 h with either piperacillin/tazobactam, tetracycline, or kanamycin antibiotic solutions, by comparing antibiotic-treated samples vs. those that were exposed to water only (see Methods). The samples consist of biofilm colonies cultivated on LBGM agar with and without (green bars) 1.5 mM CuSO_4_ (blue bars) or 0.5 mM ZnCl_2_ (gray bars). Error bars denote the s.d. as determined from at least 8 individual data points, obtained from a minimum of 2 growth batches with 2 biological replicates, and 2 technical replicates each (*B* ≥ 2, *N* = 2, *n* = 2).

In agreement with our expectation, cells retrieved from biofilms grown on LBGM agar and in the absence of Zn^2+^ or Cu^2+^ (i.e., for lotus-like superhydrophobic biofilms) show improved survival after a 1 h treatment with either piperacillin, tetracycline, or kanamycin (Fig. 5, green bars), compared to those cells from biofilms formed in the presence of metal ions. Indeed, for hydrophilic biofilms (those generated in the presence of Zn^2+^ or Cu^2+^) a ~10% increase in efficiency is observed after 1 h treatment with aqueous solutions containing piperacillin and kanamycin, compared to lotus-like biofilms (Fig. 5). The highest increase in antibiotic efficiency, that is, ~20%, is observed after a 1 h treatment with tetracycline on hydrophilic biofilms compared to lotus-like ones. Furthermore, considerable differences in final CFU/mL values are obtained when hydrophilic biofilms are treated with an antibiotic solution, compared to when they are only exposed to water for the same amount of time. In contrast, lotus-like biofilms return similar CFU/mL values after treatment with both water and antibiotic solutions. These observations indicate that the reported values are mostly a result of a chemical effect (exerted by the antibiotic) on the biofilm bacteria and are relatively independent of the sample variations and the processing of the samples.

An increased occurrence of both antibiotic resistance and bacterial persistence, in stress-rich environments, has been established, particularly for copper and zinc cations as they are generally found in the environment.^39^ Even at subtoxic levels of copper in contaminated soil, the occurrence of antibiotic resistance genes has been observed for certain bacteria.^39^ To ensure that the increased antibiotic efficiency is a direct result from the reduced protection by the biofilm matrix and does not stem from a weakening effect that acted on the bacterial cells during planktonic growth in the presence of metal ions, the same antibiotic treatment method is applied to planktonic bacteria. Here, only tetracycline is used as it showed the highest efficiency in our previous tests conducted with biofilm bacteria. Again, a similar CFU plating method is used to assess bacterial viability after the chemical challenge (see Methods). Indeed, in agreement with our expectation, treatment of planktonic bacteria with tetracycline results in no significant differences in viability—whether metal ions are present during planktonic growth or not (Supplementary Fig. 2). These results underline the importance of the physical barrier conferred by the matrix on biofilm cells and how they rely on it for protection from external stressors.

Furthermore, the antibiotic susceptibility of biofilm bacteria is also affected by the diffusive spreading of the antibiotic molecules throughout the biofilm matrix—a process that is very complex itself. Our results with fluorescently labeled WT strains show that the metal ions cause a reduced production of matrix components on the WT strain (but not a complete suppression of their expression). Thus, we expect that both the altered wetting properties of those biofilm colonies and the weakened diffusive barrier created by this altered biofilm matrix contribute to the increased susceptibility of those biofilms towards antibiotics.

### The biofilm-weakening effects can be observed also on mature superhydrophobic biofilms when CuSO_4_ and ZnCl_2_ are diffused through the substrate the biofilms are grown on

So far, we have tested if and under which circumstances the formation of superhydrophobic biofilms can be prevented by the presence of certain metal ions. However, such a strategy is only useful if the biofilms have not formed yet. The effect of metal ions on already formed biofilms has been studied mainly regarding their impact on the bulk mechanical properties, but not much on the physical surface properties of biofilms. For instance, selected metal ions have been shown to affect the viscoelastic properties of biofilms protecting them from erosion,^33,34^ and divalent ions increase the biofilm stiffness through a higher calcium carbonate content, which in turn can form diffusion barriers that shelter the inner biofilm mass.^40,41^ Indeed, the latter examples actually describe protective effects provided by the metal ions, whereas, here, the focus is on the opposite effect.

We have previously shown that altering the wetting properties of mature biofilms from superhydrophobic to hydrophilic can improve biofilm eradication methods, which involve the use of aqueous solutions. We showed that such improvements can be achieved by incubating the biofilm surfaces with concentrated solutions inducing osmotic surface dehydration effects.^2^ Motivated by those previous findings, we next assess whether the wetting resistance of already existing biofilms can also be altered if the pre-formed biofilms are further cultivated, but exposed to a liquid environment enriched with low concentrations of metal ions. For this purpose, we first generate *B. subtilis* NCIB 3610 biofilms with superhydrophobic surfaces on agar and then partially immerse the agar substrate carrying those mature (1-day-old) biofilms into liquid media containing metal ions. In other words—different from our previous study where we brought the air-exposed surface of biofilms into contact with a conditioning solution, here the metal ion-enriched medium is allowed to diffuse into the biofilm matrix via the agar substrate. Experimentally, this is achieved by dedicated, porous samples holders, which are used to carry both the agar substrate and the cultivated biofilm (Supplementary Fig. 3).

**Fig. 6:**
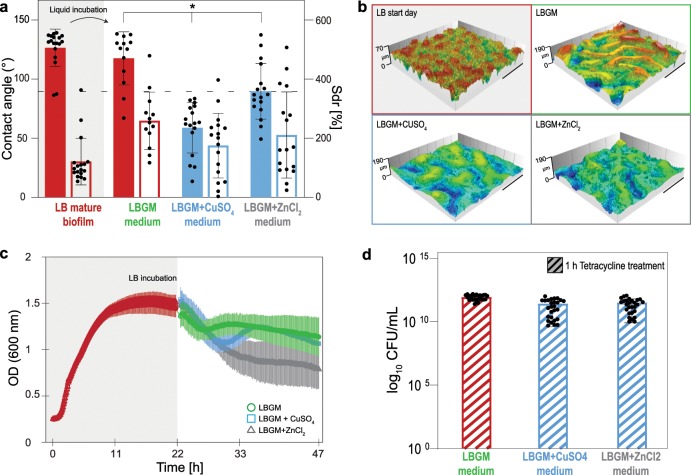
Effect of metal ions diffusing through the substrate of mature *B. subtilis* NCIB 3610 biofilms on biofilm surface wetting and topography, planktonic growth kinetics, and antibiotic efficacy. **a** Contact angle (solid bars) and Sdr (empty bars) values of initially rose-like superhydrophobic mature biofilms before and after substrate infusion with liquid LBGM medium with and without CuSO_4_ or ZnCl_2_. Asterisks denote statistically significant differences as assessed by a one-way ANOVA using *p* = 0.05 (see Methods). **b** Profilometric images of the different biofilms characterized in **a**; the scale bar represents 0.3 mm in both the *x*- and *y*-axis. **c** OD_600_ values determined for planktonic bacteria initially grown in liquid LB medium (for 22 h, red symbols) then supplemented with LBGM liquid medium with and without CuSO_4_ or ZnCl_2_. **d** Log_10_ CFU/mL values determined after a 1 h treatment with tetracycline (see Methods). The tested mature biofilms were incubated with their agar substrate infused with LBGM medium with and without CuSO_4_ or ZnCl_2_. In **a**, **d**, bar colors indicate the wetting behavior of the characterized samples (red = rose-like hydrophobic; blue = hydrophilic). Error bars denote the s.d., which was determined as follows: **a** from at least 13 individual data points, obtained from 6 growth batches with a minimum of 2 biological replicates, and 1 technical replicate each (*B* = 6, *N* ≥ 2, *n* = 1); **c** from a total of 96 individual data points, obtained from 3 growth batches with 1 biological replicate, and 32 technical replicates each (*B* = 3, *N* = 1, *n* = 32); **d** from a total of 27 individual data points, obtained from 3 growth batches with 3 biological replicates, and 3 technical replicates each (*B* = 3, *N* = 3, *n* = 3).

Before determining the influence of metal ions on the wetting properties of biofilms, we verify that the transfer procedure itself, that is, growing the mature biofilms for another 24 h, does not alter their wetting behavior. Pre-tests show that for rose-like hydrophobic biofilm samples grown on LB agar, a transfer into LBGM medium is necessary to maintain their rose-like wetting properties. Since at those conditions the biofilm keeps growing, the biofilm surface complexity is increased during this additional day of incubation (Fig. 6a). The same change in nutrient media, and thus in growth conditions, is then also applied to planktonic bacteria. Although a slight decline in the growth curves is observed when the metal ions are added, the remaining OD_600_ is still reasonably high (Fig. 6c).

When initially rose-like mature biofilms are further cultivated in LBGM medium containing either 1.5 mM CuSO_4_ or 0.5 mM ZnCl_2_, the biofilms become wettable with contact angles <90° (Fig. 6a). At the same time, those hydrophilic biofilms—compared to the ones that are further cultivated in standard LBGM—show reduced surface roughness, which can be attributed to the presence of the metal ions (Fig. 6a, b). Again, a high variability in both the measured contact angle and Sdr values is observed for mature biofilms exposed to ZnCl_2_-containing LBGM liquid medium: some samples behave slightly hydrophobic and others strongly hydrophilic (Fig. 6a). Yet, for all samples exposed to the metal ions, their superhydrophobic properties disappear and—on average—their surface features are considerably lower than for the control samples (Fig. 6a, b). This shows that the effect of CuSO_4_ and ZnCl_2_ on preventing the formation of superhydrophobic biofilm surfaces is not limited to newly formed biofilms, but can also occur when a mature biofilm has already been established.

In contrast, when the substrate of mature, hydrophilic biofilms (i.e., such biofilms that are cultivated in the presence of metal ions) is exposed to LBGM liquid media devoid of metal ions overnight, the colonies do not show major changes in their surface characteristics. The contact angle and Sdr values are maintained or only slightly increased for both types of biofilms: those grown in the presence of copper or zinc (Supplementary Fig. 4). These results indicate that the effect of the metal ions on the biofilm matrix and, in turn, on the biofilm surface roughness and wetting are not easily reversible—at least not by diffusing a nutrient rich medium through their substrate overnight.

Finally, we also test the efficacy of an antibiotic solution on mature biofilms whose surface wetting properties are tuned from rose-like superhydrophobic to hydrophilic. For this purpose, we chose again the antibiotic that showed the strongest efficiency in the previous tests, that is, tetracycline. Also here, the entire treated biofilm mass is harvested from the substrate and exposed to an aqueous solution containing 1.28 mg/mL of tetracycline for 1 h before conducting CFU testing. The initial conditions (e.g., before the antibiotic treatment) of the biofilm colonies do not greatly differ in terms of size or weight (see Methods). Therefore, in this case (unlike in the previous section), the effect of the antibiotic treatment is reported directly as cell survival and is depicted in log_10_ CFU/mL values (Fig. 6d). It is observed that when metal ions are added to the liquid LBGM medium, onto which the mature biofilms are transferred, two effects can be identified. First, the biofilm surfaces become hydrophilic, and second, after a chemical challenge, a lower cell survival is observed compared to the hydrophobic rose-like biofilms (Fig. 6d). Control experiments, using water instead of an antibiotic solution, are performed also on those type of samples, for example, mature biofilm colonies whose substrate is diffused with liquid medium (with and without metal ions) overnight. After a 1 h exposure to water, all biofilm colonies show similar CFU/mL values, even those that were exposed to metal ions (Supplementary Fig. 5). The previous indicates that before the antibiotic treatment, a comparable amount of viable cells are found in the differently treated biofilms, and that the effect seen in Fig. 6d stems mostly from the antibiotic treatment.

Pre-conditioning approaches, that is, treatment of the material before the use of antibiotics, can be a promising strategy for enabling a complete eradication of biofilms; other studies have shown that the effect of antibiotics can be enhanced in different ways: that is, by using electrochemical scaffolds on chronic wounds^42^ or by disrupting the EPS biopolymers.^25^ Furthermore, not only chemical but also mechanical attack can be facilitated by pre-conditioning, as an enhanced biofilm erosion has been reported for hydrophilic and/or smooth biofilms compared to rough and/or hydrophobic ones.^2,43^

In conclusion, here, we showed that the presence of the metal cations Cu^2+^ or Zn^2+^ during the cultivation of bacteria induced changes in the resulting biofilm matrix, and the said changes altered the biofilm surface in such a way that otherwise superhydrophobic biofilms become completely wettable. Furthermore, the same low concentrations of metal ions induced very similar effects on mature biofilms when they were diffused through the substrate the biofilms are grown on. In both cases, an increased bacterial inactivation was observed when the entire biofilm material was treated with aqueous solutions containing antibiotics.

Abolishing or at least weakening the superhydrophobic properties of biofilms can be an important step in fighting such sturdy materials: if successful, it allows for a more efficient accessibility of the embedded bacteria towards mechanical or chemical attack. Of course, finding a one-step, gold standard solution that is able to eliminate all kinds of biofilms would be optimal; yet, considering the broad variety of biofilm-forming microorganisms and the large differences in the molecular composition of these biofilms, this appears unrealistic. Thus, a multi-faceted approach combining inhibition, pre-conditioning, inactivation, and/or dispersal steps is likely to be the most efficient strategy to control biofilms. The method presented here introduces such a pre-conditioning strategy, which targets a physical property of biofilms, that is, their wetting resistance. Our results show that low concentrations of metal ions are sufficient to disturb the production of biofilm matrix components, and that this effect unspecifically targets different biomolecules at the same time. This indicates that the approach introduced here could also be applicable to biofilms generated by other bacterial species, such as the pathogenic microorganism *Vibrio cholerae*, which has been shown to form biofilms with liquid-repellent surfaces.^44^ As the metal ions can be applied to biofilms in the form of an aqueous solution, it should be possible to target different forms of biofilms at different stages of their development and in different growth environments.

## Methods

### Bacterial strains

*Bacillus subtilis* NCIB 3610 was obtained from the lab of Roberto Kolter. Naturally competent derivative of strain NCIB 3610, DK1042,^45^ was used to probe promoter activities, including strains TB34 P_hyperspank_-*gfp*,^46^ TB363 P_*eps*_-*gfp*,^47^ TB373 P_*tapA*_-*gfp*,^47^ and TB685 P_*blsA*_-*gfp*.^48^

### Bacteria cultivation in liquid media

For all strains, liquid overnight (O/N) cultures were prepared as follows: 10 mL of sterile 2.5% (w/v) lysogeny broth or LB medium (Luria/Miller, Carl Roth, Karlsruhe, Germany) were inoculated with a frozen bacterial/glycerol stock. Then, the bacterial solution was incubated at 37 °C and 90 r.p.m. in a shaking incubator (Sartorius, Göttingen, Germany) overnight.

### Biofilm cultivation in semi-solid media

Two different base growth media were used for cultivation of the different *B. subtilis* strains: first, standard 2.5% (w/v) LB medium (Fisher Scientific, New Hampshire, USA); second, LBGM medium, that is, 2.5% (w/v) LB medium (Luria/Miller, Carl Roth, Karlsruhe, Germany) enriched with 100 μM manganese(II)sulfate (MnSO_4_) and 1% (v/v) glycerol.^32^ The different culture media were mixed with 1.5% (w/v) agar-agar (Carl Roth, Karlsruhe, Germany), autoclaved, and stored in the oven at 60 °C to maintain a constant temperature. To generate semi-solid nutrient layers for biofilm growth, ~22 mL of the media-enriched liquid agar were pipetted into petri dishes and allowed to cure for 30 min with the lid half open under laminar flow. Addition of metal ions into the two media types was performed at 60 °C from sterile stock solutions of 150 mM CuSO_4_ or 50 mM ZnCl_2_, respectively. To obtain bacterial biofilm colonies, three separate 5 μL drops of bacterial liquid culture were pipetted onto each petri dish and cultivated upside down at 30 °C for 24 h.

The concentrations of the metal ions were chosen based on the following considerations: we aimed for metal ion concentrations that are high enough to possibly induce changes in biofilm formation (and affect biofilm wetting), but low enough to still allow the bacteria to reproduce. Hence, in preliminary experiments (a re-growth assay as that performed by Grumbein et al.^33^), we tested metal ion concentrations in LB medium ranging from 0 mM (control) to 10 mM (maximal concentration). The concentrations presented in this manuscript were found to allow the bacteria to still reach similar OD_600_ values in the presence of the metal ions as in the control groups, where no metal ions were added. Conversely, when NCIB 3610 bacteria were incubated in LB containing 10 mM of either CuSO_4_ or ZnCl_2_, OD_600_ values close to zero were obtained. Biofilm formation was not tested at this elevated concentration of metal ions as we assumed that—due to the high level of toxicity in the substrate—the bacteria would not be able to form a biofilm. It is, of course, possible that higher concentrations than those chosen by us, that is, concentrations between 1.5 and 10 mM, could still meet the aforementioned aim; however, it has been shown earlier that CuSO_4_ and ZnCl_2_ can cause changes in the material properties of certain biofilms.^33,34^ For instance, the elastic modulus of biofilms formed by the strain *B. subtilis* B-1 is increased by almost 2 orders of magnitude when the biofilms are exposed to either CuSO_4_ or ZnCl_2_.^33^ As the main goal of the study was to observe the impact of metal ions on the physical properties of biofilms, special care was taken to avoid such concentrations of metal ions that are likely to strongly impact the mechanical properties of this biomaterial.

### Bacterial growth kinetics

Bacterial growth curves (Figs. 1d, e, 2a, 3a, and 6c) were determined with a plate reader (POLARstar OPTIMA, BMG Labtech). Overnight cultures of *B. subtilis* WT, P_hyperspank_-*gfp*, P_*tapA*_-*gfp*, P_*bslA*_-*gfp*, and P_*eps*_-*gfp* were generated as described above and diluted in 200 µL of the desired liquid medium to an OD_600_ of 0.05. Both LB and LBGM^32^ were used as base media. Planktonic bacterial growth was followed for ~24 h, while the cultures (in 96-well plates) were maintained at constant shaking at 37 °C. Optical density was measured at 600 nm every ~7 min. The resulting growth curves represent the mean of all individual wells (referred to by *n* and specified in the respective figure captions) on different growth days (referred to by *B* and specified in the respective figure captions).

For the growth curves shown in Fig. 6c, overnight cultures of *B. subtilis* WT were generated, diluted, and bacterial growth was followed as described above. However, the measurement was paused after ~22 h, at which point the cultures were removed from the plate reader and challenged with the different metal ions under sterile conditions. For this, 20 µL of LBGM liquid medium was added to the control samples, amounting for 10% of the total volume. The same volume of LBGM containing either CuSO_4_ or ZnCl_2_ was added to the rest of the wells to obtain final concentrations of 1.5 and 0.5 mM, respectively. Afterwards, the growth kinetics were followed for another ~24 h.

The effect of metal ions on the metabolic activity of the bacteria was also assessed for planktonic WT *B. subtilis* when cultivated in the presence of metal ions. An ATP determination kit (Thermo Fisher, MA, USA) was used to measure concentrations of this molecule in planktonic bacteria using a Victor^3^ (PerkinElmer, MA, USA) plate reader to detect luminescence. The samples comprised overnight cultures (prepared as described above) containing 1.5 mM CuSO_4_ or 0.5 mM ZnCl_2_ in the LB medium and standard LB medium as a control. Extracellular ATP was measured at stationary growth phase of the cultures following the manufacturer’s instructions. With this assay, no significant difference was found between the different samples (Supplementary Fig. 6).

### Treatment of mature biofilms with metal ions

Mature biofilm colonies were generated as described above with the difference that, here, 15 mL LB agar plates were used to obtain a thinner substrate. LBGM liquid medium was prepared as indicated above and autoclaved; once cooled down, the metal ions were added to a final concentration of 1.5 mM CuSO_4_ or 0.5 mM ZnCl_2_, respectively; a fraction of LBGM was left unaltered and used as a control. Diffusion of liquid medium into the agar substrate of the biofilms was established using the set-up illustrated in Supplementary Fig. 3. In brief, the procedure was as follows: first, 7 mL of the liquid medium were pipetted into each well of a 6-well plate; then, the polytetrafluoroethylene (PTFE) sample holders were tightly fitted all the way down into each well; last, ~1.5 mL of liquid media were pipetted into each well (through the holes in the surface of the sample holders) until the liquid would protrude from the surface to allow for contact with the agar substrate (Supplementary Fig. 3). The mature biofilm colonies and their underlying agar substrate were extracted from the petri dishes by cutting the agar around them using a cookie cutter with a diameter of 3 cm and a spatula for lifting. Then, the individual biofilm colonies were carefully placed onto the PTFE sample holders while making sure that all the holes on the surface of the sample holder were covered. To avoid abrupt changes in humidity during incubation, the well plates (covered with a lid) were placed inside a sterile plastic bag before placing them into the incubator at 30 °C for ~20–24 h. Changes in the biofilm surface properties were observed for incubation times as short as ~18 h, but with quite variable results: at those shorter incubation times, some samples showed changes, while others did not. We attributed this to variable diffusion kinetics among the samples due to small differences in the sample holders (handmade pieces) and the sample preparation. Therefore, we decided to use slightly longer incubation times, which yielded more uniform and well-reproducible results.

### Contact angle measurements

To probe the wetting behavior of the different biofilm variants, a 10 μL droplet of ddH_2_O was placed onto the biofilm surface, and a transversal image of the liquid–solid interface was captured using a high-resolution camera (Point Gray Research, Richmond, Canada). The static contact angle value was determined using the software ImageJ and the “drop snake” plug-in. Afterwards, superhydrophobic biofilm samples (i.e., those with static contact angles >120°) were tilted and the response of the liquid droplet was observed to distinguish between rose-petal (high adhesion: droplet sticks) and lotus-like (low adhesion: droplet rolls off) hydrophobicity. Rose-like hydrophobicity is indicated throughout the figures with red color, whereas lotus-like hydrophobicity is indicated with green.

### Topographical characterization

To investigate the topographical changes on the biofilm surfaces, light profilometry images were acquired using a Nanofocus μsurf profilometer (NanoFocus AG, Oberhausen, Germany). Pictures were acquired at ×20 magnification producing surface images with an area of 800 × 772 μm^2^. The topographical data was evaluated with the software μsoft (Version 6.0, NanoFocus AG, Oberhausen, Germany). Only images with a minimum of 60% measured data points were considered for analysis, and missing data points were interpolated. From those topographical profiles *z*(*x*, *y*), the developed interfacial area ratio, ${\mathrm{Sdr}} = \frac{1}{A}\left[ {\mathop {\iint}\nolimits_A {\left( {\sqrt {\left[ {1 + \left( {\frac{{\partial z(x, \, y)}}{{\partial x}}} \right)^2 + \left( {\frac{{\partial z(x, \, y)}}{{\partial y}}} \right)^2} \right]} - 1} \right)} {\mathrm{d}}x{\mathrm{d}}y} \right]$, was calculated. This metrological parameter is defined in the ISO 25178 norm, which specifies terms, definitions, and parameters for the determination of surface texture by areal methods;^49^ throughout the text, this parameter is referred to as the “Sdr value”. The resolution of the images was 1.56 μm in lateral direction. The step size in *z* direction was 0.22 μm; however, owing to the peak detection algorithm, the profilometer uses the resolution in *z* is better than this step size and can—under ideal conditions—be as good as 10 nm with the objective used here.

### Fluorescence microscopy

Entire biofilm colonies were analyzed using an Axio Zoom V16 stereomicroscope (Carl, Zeiss, Jena, Germany) equipped with a Zeiss CL 9000 LED light source and an AxioCam MRm monochrome camera (Carl Zeiss). Images were captured at ×5 magnification using both wide-field and fluorescence mode; for the latter, the HE eGFP filter set #38 (Carl Zeiss, excitation at 470/40 nm and emission at 525/50 nm) was used to image GFP fluorescence. Relative fluorescence mode values were calculated by subtracting the fluorescence mode of each biofilm colony from its respective (average) background fluorescence mode. For each growth day (*B* = 3), an average background fluorescence mode was calculated from a minimum of two WT biofilm colonies imaged with the GFP filter. Throughout the manuscript, relative fluorescence intensity mode values are referred to as “fluorescence intensity values” for simplicity.

The fluorescence of purified GFP (Merck, Darmstadt, Germany) mixed with solutions of 1.5 mM CuSO_4_ or 0.5 mM ZnCl_2_ was analyzed both at acidic and neutral pH using a Victor^3^ plate reader (PerkinElmer, MA, USA). Neutralization of the solutions containing GFP was performed using Tris buffer and NaOH.

### Antibiotic efficacy tests and determination of CFUs

As there are no established MIC values reported for the specific combination of bacterial strains and antibiotics used in this study, the concentration of the antibiotics was determined based on published MIC_90_ values for different *Bacilli*. An MIC_90_ of 1 μg/mL is reported for piperacillin/tazobactam (1:8), a range between 0.032 and 8 µg/mL is reported for tetracycline^50–52^ and a value of 8 µg/mL for kanamycin.^51^ Therefore, a concentration of 8 µg/mL was used for the treatment of planktonic bacteria with tetracycline. For the treatment of biofilm bacteria, piperacillin/tazobactam (8:1) was used at a concentration of 0.15 mg/mL and both tetracycline and kanamycin were used at a concentration of 12.8 mg/mL (this is ~150× higher than the MIC values reported in the literature). The reason for selecting such elevated concentrations was that biofilms can be 100–1000× more resistant than planktonic bacteria.^53^ Therefore, antibiotic concentrations should be significantly higher for biofilms than for planktonic bacteria to avoid the risk of using insufficient levels that induce antibiotic resistance.^54^ Although testing antibiotics on non-pathogenic bacteria is not common practice, we present this method as ground work for subsequent tests on pathogenic microorganisms.

To test the efficiency of the three different antibiotics on biofilms, entire bacterial colonies were scraped from the underlying agar, pooled, and suspended in 1 mL of an antibiotic solution prepared in ddH_2_O. In parallel, additional bacterial colonies were also incubated in pure ddH_2_O for the same amount of time and subjected to the same subsequent methodology—serving as controls. The biofilm suspensions were left undisturbed at room temperature (RT) for 1 h; afterwards, the biofilm suspensions were centrifuged at 5000 r.p.m. and 5 °C for 20 min. The pellet was washed twice (using 1 min centrifugation steps between the washing steps) with a saline solution (prepared by dissolving 9 g of NaCl in 1 L ddH_2_O) and then resuspended. For biofilms grown in the presence or absence of metal ions, the following specifications were used: hydrophobic biofilms were resuspended in 10 mL saline solution, whereas hydrophilic ones in 5 mL (as the mass of these colonies were approximately half of the hydrophobic ones: ~30 vs. ~15 mg). For mature hydrophobic biofilms that were subjected to diffusion of LBGM media through their substrate (with or without metal ions) before the antibiotic treatment, all samples types were resuspended in 10 mL saline solution as their mass did not differ greatly (~400 mg). To obtain a homogeneous bacterial cell suspension, a SONOPULS ultrasonic homogenizer (BANDELIN, Berlin, Germany) was used at a frequency of 20 kHz and 20% amplitude using 1 pulse/s. For biofilms grown in the presence or absence of metal ions, the following specifications were used: a duration of 1 min 48 s of ultrasonic pulses was used for samples containing hydrophobic biofilms, whereas 1 min of ultrasonic pulses was used for hydrophilic biofilms. For mature hydrophobic biofilms that were subjected to diffusion of LBGM media through their substrate (with or without metal ions), all samples types were subjected to ultrasonic pulses with a duration of 1 min 48 s. OD_600_ values were determined for the resulting cell suspensions to ensure that loss of material (e.g., cells) was minimal. Then, the cell suspensions were serially diluted, and 100 µL of the dilution product was inoculated on agar plates in duplicates (using two dilution factors separately). CFUs were assessed after incubation of the agar plates at RT for 3 days or at 37 °C for 24 h. A schematic illustrating the procedure is included in the Supplementary Fig. 7.

Antibiotic treatment of planktonic bacteria (cultivated in the presence and absence of metal ions) was conducted using a modified version of the method reported by Nuno Cerca et al.^55^ Briefly, O/N cultures (prepared in either LB, LB + 1.5 mM CuSO_4_, or LB + 0.5 mM ZnCl_2_, as stated above) were diluted to an OD_600_ of 0.05 and incubated at 37 °C and 90 r.p.m. until an OD_600_ of 0.1 was reached (“day culture”). Then, the day culture was centrifuged at 5000 r.p.m. and 5 °C for 5 min, the pellet was washed twice (as described above) and resuspended to an OD_600_ of 0.05 in fresh LB containing the antibiotic. The resulting suspensions were further incubated at 37 °C and 90 r.p.m. for 1 h. After the antibiotic treatment, the samples were again centrifuged, washed, and resuspended in saline solution to an OD_600_ of 0.05 before serial dilutions were generated and plating was conducted. Three dilution factors were selected and plating was performed in triplicates to assess CFUs.

For the determination of CFUs from O/N cultures of NCIB 3610 bacteria incubated in the presence and absence of metal ions, the following method was used. O/N cultures were prepared in either LB, LB + 1.5 mM CuSO_4_, or LB + 0.5 mM ZnCl_2_ (as stated above) and incubated at 37 °C and 90 r.p.m. for 18 h (following the same conditions as used for the rest of the experiments in the paper). OD_600_ values were determined, and a day culture was prepared by diluting the O/N culture in 5 mL (of their respective) fresh medium to a final OD_600_ of 0.05. These day cultures were then further incubated for 1–2 h until an OD_600_ of 0.1 was reached. Then, the day cultures were centrifuged at 5000 r.p.m. for 10 min, and washed and resuspended as stated above. OD_600_ values were determined in saline solution as well to ensure that loss of materials (e.g., cells) was minimal. Finally, serial dilutions were performed, and three dilution factors were selected to be plated in triplicates to assess CFUs.

For all tests, CFU/mL values were obtained by multiplying the average CFU calculated from the replicates by the dilution factor, divided by the inoculation volume. Efficiency of the antibiotic treatment was assessed by subtracting the values (log_10_ CFU/mL) obtained from samples treated with water only, from those obtained from samples treated with an antibiotic solution. The result from this subtraction was divided by the first value (samples treated with water) and multiplied by 100 to obtain percentage values.

### Statistical analysis

Sample sizes are described throughout the manuscript as follows: for growth batches (e.g., biological samples generated on different days), the symbol *B* is used; for independent biological replicates (e.g., biofilm colonies generated on the same day), the symbol *N* is used, whereas for technical replicates, the symbol *n* is used. A minimum of two growth batches (*B* ≥ 2) and two independent biological replicates (*N* ≥ 2) were deemed as appropriate to obtain reproducible results in all tests of this work (supported with preliminary tests). Similarly, when technical replicates were possible, a minimum of two growth batches (*B* ≥ 2) with a minimum of one biological replicate (*N* ≥ 1), and a minimum of two technical replicates each (*n* ≥ 2), were deemed as appropriate to obtain reproducible results. Statistically significant differences between biofilms grown in standard nutrient media and those grown on or treated with metal ions were assessed for Sdr, contact angle, and fluorescence intensity values using a one-way analysis of variance (ANOVA) when samples met the assumption of homogeneity of variances (as verified by Levene test), followed by a Tukey’s post hoc test. A Welch ANOVA followed by Game–Howell post hoc was used to assess samples whose datasets failed the assumption of homogeneity of variances. For all datasets, normality was tested with a Shapiro–Wilk test, and (less confining) by *q–q* plots and histograms (as large variability is expected within biological samples). To determine statistical significance, normality, and homogeneity of variances, a *p* value of 0.05 was used for all tests reported in this work.

### Fluorescence signal at the single-cell level

To monitor the effect of metal ions at the single-cell level, bacterial biofilm colonies were generated as described above. After 24 h of incubation, colonies were sonicated in NaCl 0.9% individually, then 5 µL of this sonicated culture was spotted onto a microscope slide coated with 1% agarose, covered with a coverslip and examined under the fluorescence microscope (Olympus Bx51; ×100 oil objective). Images were captured using bright light (exposure time 50 ms) and fluorescence light (using the GFP filter exposure time 500 ms). For details on the data analysis, see Supplementary Fig. 8.

### Reporting summary

Further information on research design is available in the Nature Research Reporting Summary linked to this article.

## Supplementary information

Supplementary Information

Reporting Summary Checklist

## Data Availability

The datasets generated during the current study are available from the corresponding author on reasonable request.

## Code availability

No relevant code was generated during the current study.
