# Supplementary material for: Metal ions weaken the hydrophobicity and antibiotic resistance of Bacillus subtilis NCIB 3610 biofilms
Source: NPJ Biofilms Microbiomes. 2020 Jan 3;6:1. doi: 10.1038/s41522-019-0111-8 (PMC6941983; doi:10.1038/s41522-019-0111-8)
Supplement: Supplementary file 1 — Supplementary Information [file 41522_2019_111_MOESM1_ESM.pdf]

Supplementary Information for:

Effect of metal ions on *Bacillus subtilis* NCIB 3610 biofilm surface hydrophobicity and susceptibility towards antibiotics

Carolina Falcón García[1], *Martin Kretschmer*[1], *Carlos N. Lozano-Andrade*[2], *Markus Schönleitner*[1], *Anna Dragoŝ*[2], *Ákos T. Kovács*[2], *and Oliver Lieleg* [1,#]

[1]Department of Mechanical Engineering and Munich School of Bioengineering, Technical University of Munich,
Boltzmannstraße 11, 85748 Garching, Germany

[2] Bacterial Interactions and Evolution Group, Department of Biotechnology and Biomedicine, Technical University of Denmark
Søltofts Plads 221, 2800 Kgs. Lyngby

[#] Corresponding author:

Prof. Dr. Oliver Lieleg
Department of Mechanical Engineering and Munich School of Bioengineering, Technical University of Munich,
Boltzmannstraße 11, 85748 Garching, Germany
e-mail: oliver.lieleg@TUM.de, phone: +49 89 289 10952, fax: + 49 89 289 10801

Keywords: biofilm, hydrophobicity, topography, metals, EPS, antibiotics, GFP

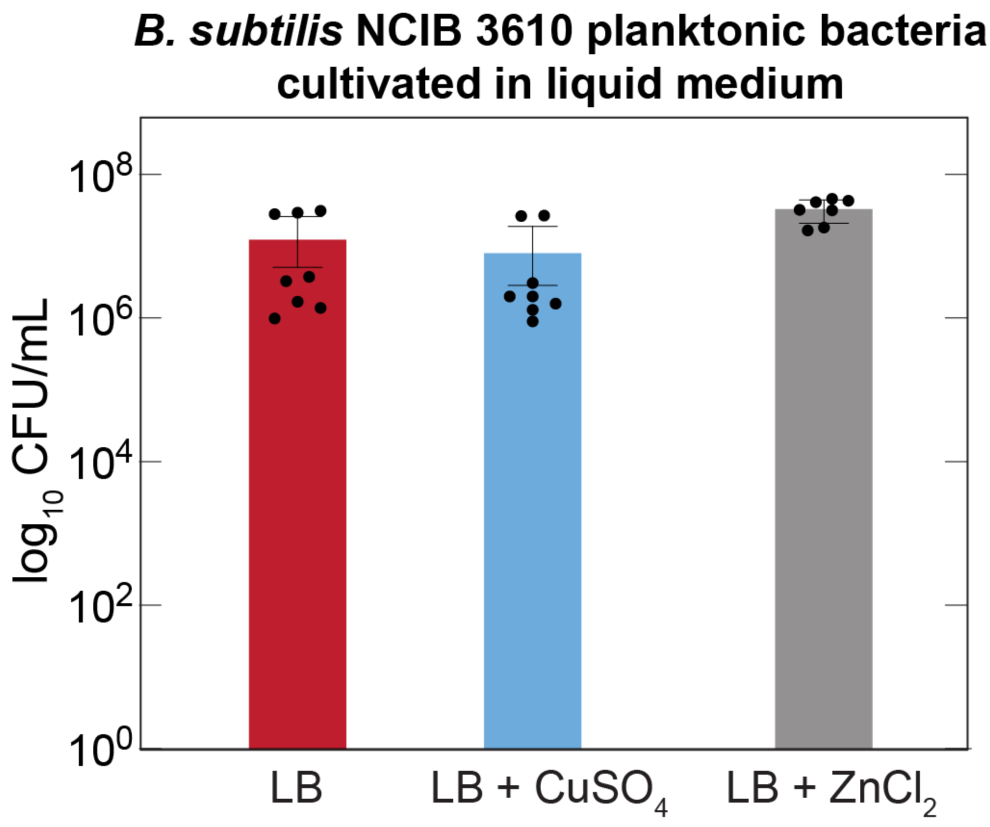

Supplementary Figure 1. **Log$_{10}$ CFU/mL values of planktonic *Bacillus subtilis* NCIB 3610 bacteria cultivated in LB liquid medium with and without metal ions.** LB was supplemented with 1.5 mM $CuSO_4$ (blue bar) and 0.5 mM $ZnCl_2$ (grey bar), respectively. Error bars denote the standard deviation (s.d.) as determined from at least 7 individual data points obtained from 3 growth batches with 1 biological replicate, and a minimum of 2 technical replicates each ($B = 3$, $N = 1$, $n \geq 2$).

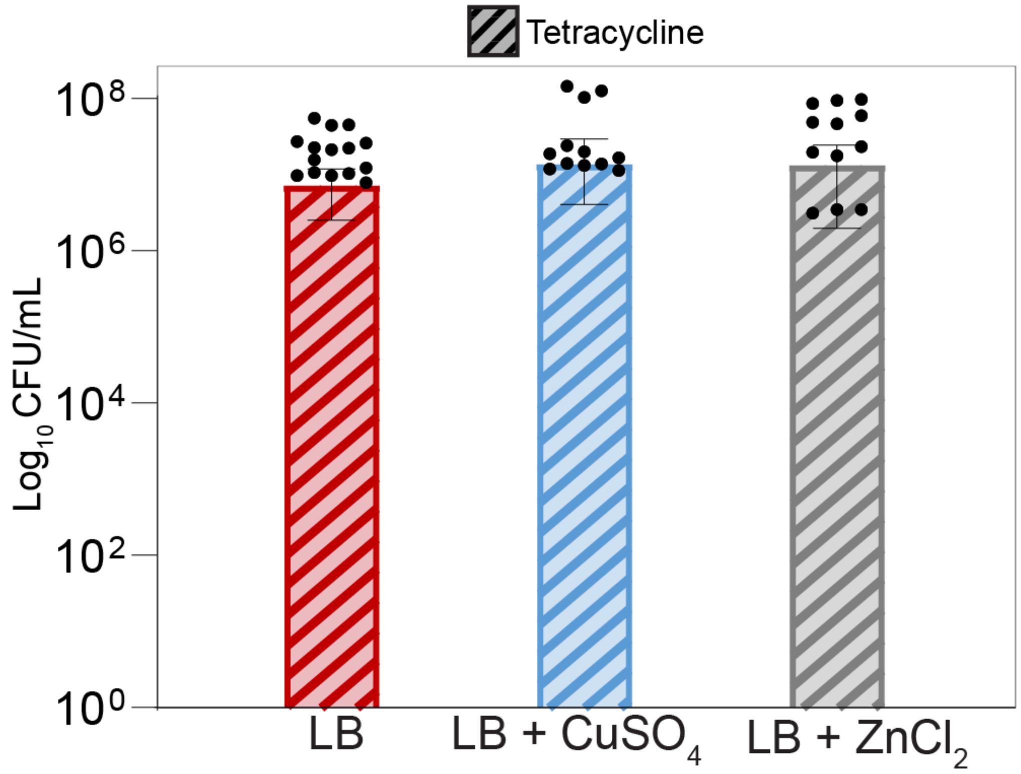

***Supplementary Figure 2*. Log$_{10}$ CFU/mL values after 1 h treatment with Tetracycline (8 µg/mL) on *B. subtilis* NCIB 3610 planktonic bacteria cultivated in LB liquid medium with and without metal ions.** LB was supplemented with 1.5 mM $CuSO_4$ (blue bar) and 0.5 mM $ZnCl_2$ (grey bar), respectively. No statistically significant differences were found among the samples, as assessed by a one-way ANOVA (see Methods) using a p-value of 0.05. Error bars denote the s.d. as determined from at least 12 individual data points obtained from a minimum of 4 growth batches with 1 biological replicate, and 3 technical replicates each ($B \geq 4$, $N = 1$, $n = 3$).

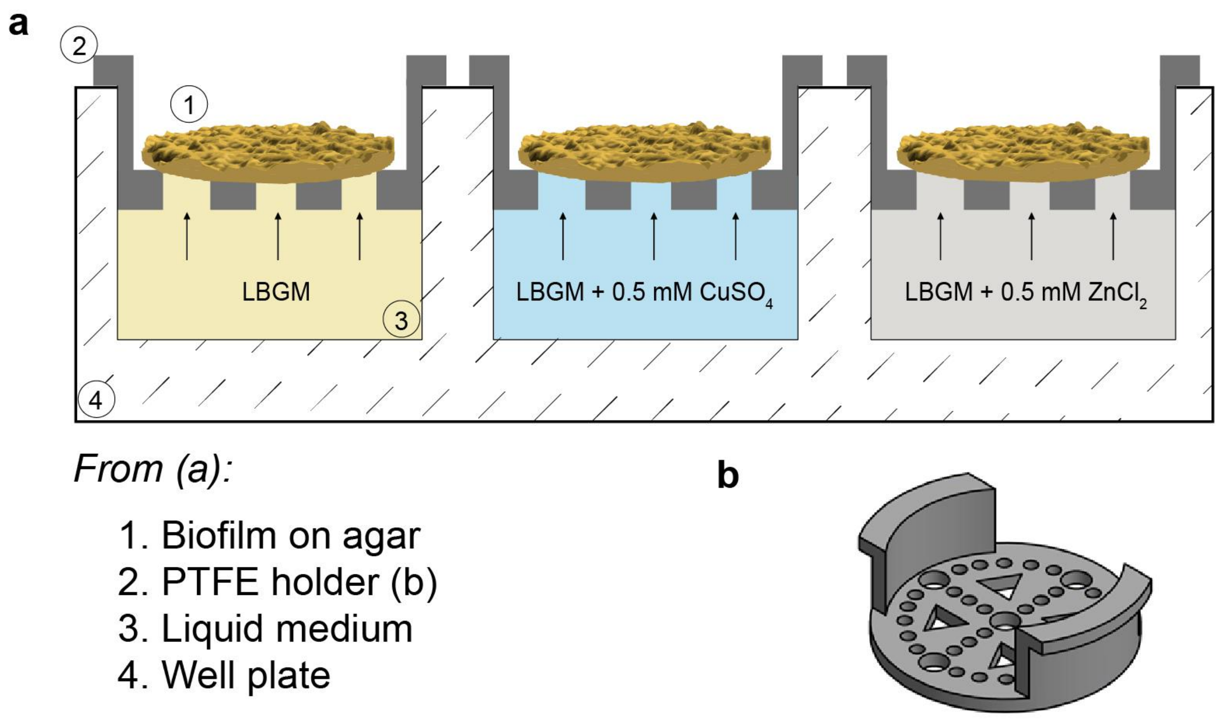

***Supplementary Figure 3*. Treatment of mature biofilm samples. a**, schematic depicting the set-up employed for the treatment of mature *B. subtilis* NCIB 3610 biofilms with LBGM liquid medium with and without metal ions. **b**, PTFE sample holder used in the set-up from **(a)**.

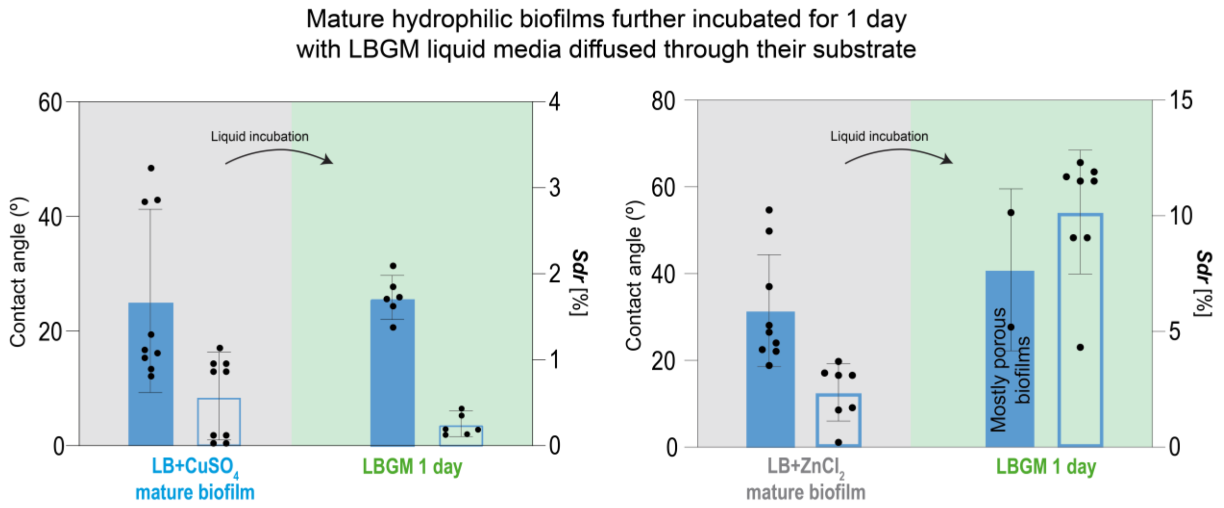

Supplementary Figure 4. **Mature hydrophilic biofilm colonies further incubated overnight with LBGM liquid medium diffused through their substrate cannot recover their original hydrophobic behavior.** Contact angle (closed bars) and *Sdr* values (open bars) of *Bacillus subtilis* NCIB 3610 biofilm colonies grown on LB + 1.5 mM $CuSO_4$ **(a)** or LB + 0.5 mM $ZnCl_2$ **(b)** before and after diffusion of LBGM liquid medium through their agar substrate overnight. Data before liquid diffusion corresponds to that presented in Fig. 1a of the main text. For data after liquid diffusion; error bars denote the s.d. as determined from at least 6 individual data points obtained from 2 growth batches with a minimum of 2 biological replicates, and 1 technical replicate each ($B = 2$, $N \geq 2$, $n = 1$).

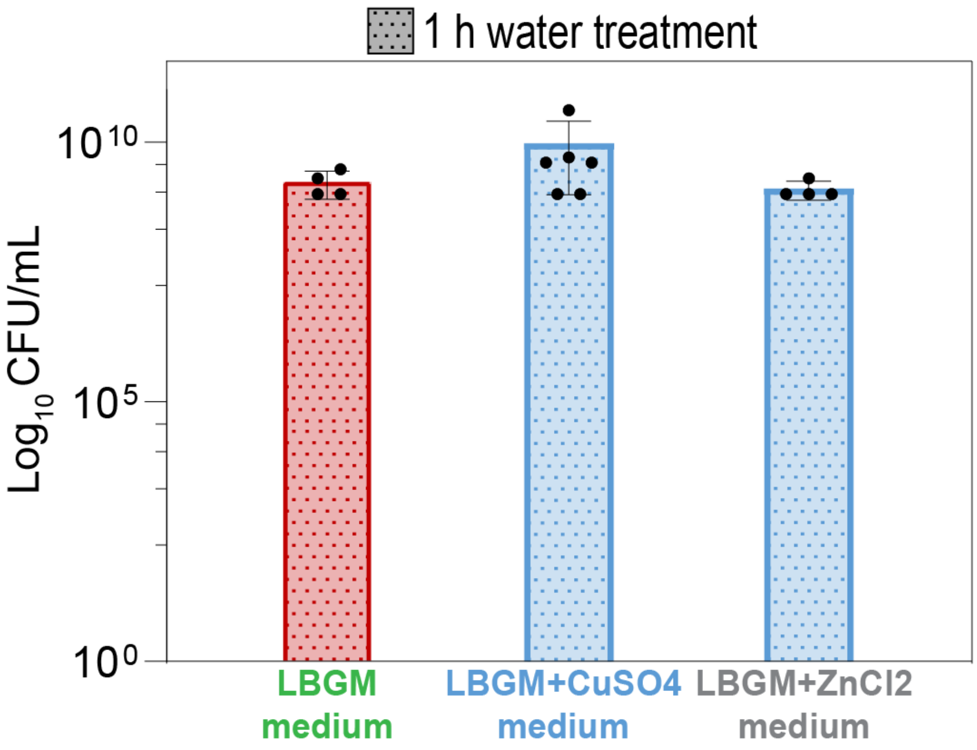

Supplementary Figure 5. **Water treatment on mature hydrophobic biofilms whose substrate was diffused with LBGM liquid medium both, with and without metal ions.** Control experiment for those samples presented in Fig. 6d of the main text. Instead of a 1 h incubation in aqueous solutions containing Tetracycline, the biofilm colonies were incubated only in water for the same amount of time. Log_{10} CFU/mL values show a similar amount of viable cells in the three samples types after the water treatment. The previous suggests that the initial conditions before the antibiotic treatment shown in Fig. 6d are similar. The color of the bars represent the wetting behavior of the samples (red = rose-like hydrophobic, blue = hydrophilic). Error bars denote the s.d. as determined from at least 4 individual data points obtained from 2 growth batches with a minimum of 1 biological replicate, and a minimum of 2 technical replicates each ($B = 2$, $N \geq 1$, $n \geq 2$).

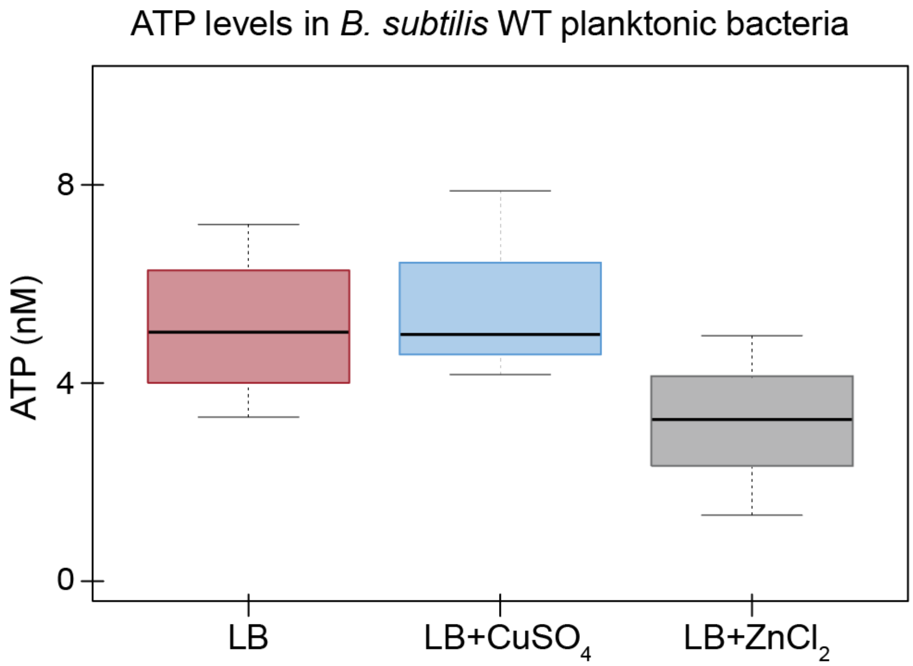

***Supplementary Figure 6*. Effect of metal ions on extracellular ATP of planktonic *B. subtilis* NCIB 3610 bacteria.** No statistical significant differences were found among the different samples, as assessed by a one-way ANOVA using a *p*-value of 0.05. Boxes represent the first quantile and the third quantile, lines represent the median, and the whiskers span from the maximum to the minimum value ($B \geq 3$, $N = 1$, $n \geq 2$).

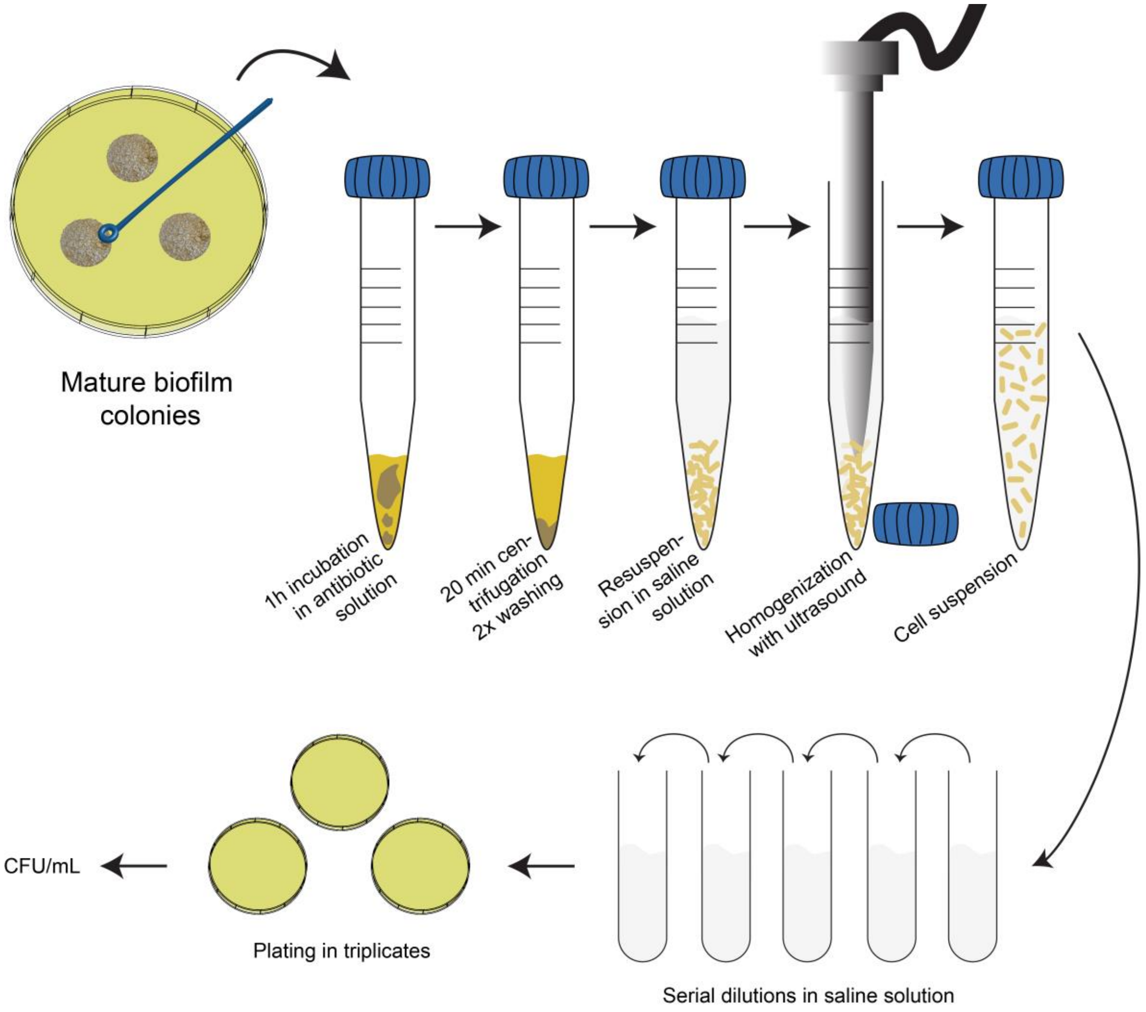

Supplementary Figure 7. **Method employed for testing antibiotic efficiency of mature biofilm colonies.** CFU plating method was used to account for cell viability. Different dilution factors were used based on the type of sample.

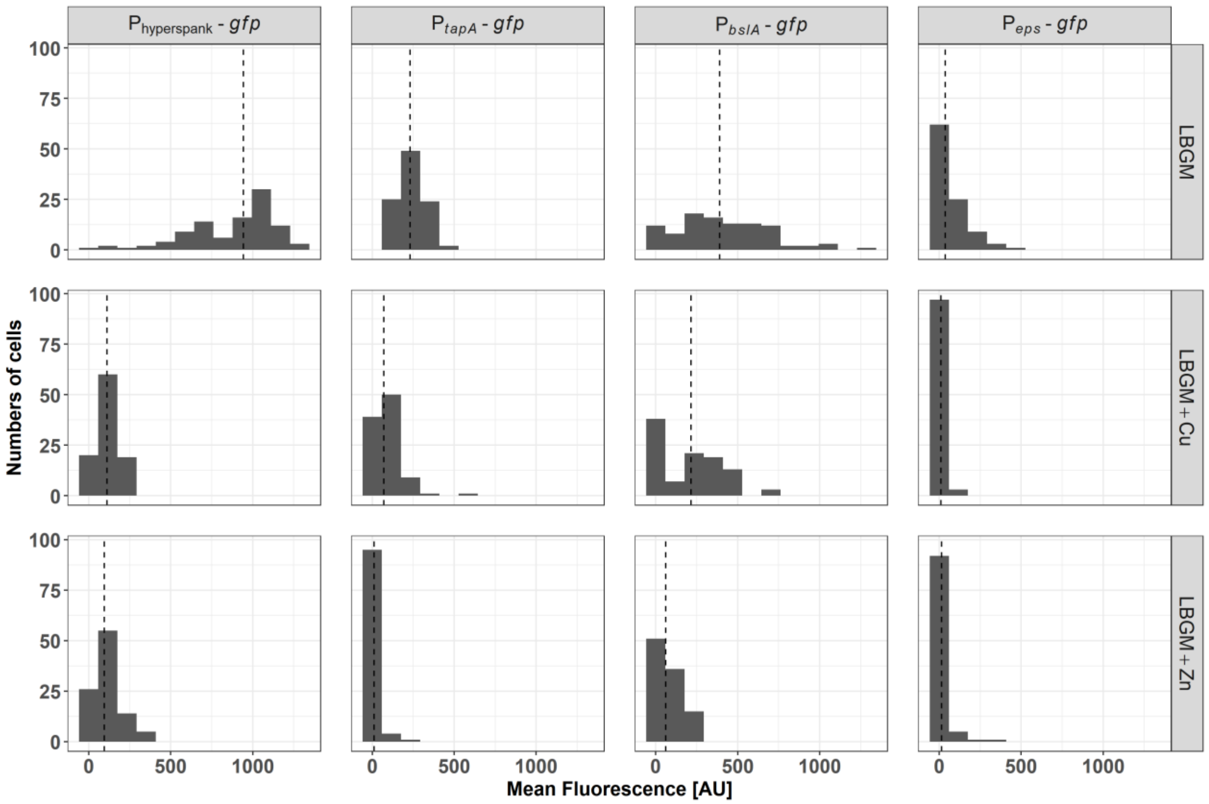

Supplementary Figure 8. **Effect of metal ions on *Bacillus subtilis* NCIB 3610 at the single-cell level.** Expression from the reporters detected at the single-cell level. Histograms were created after randomly selecting 100 bacterial cells and detecting mean fluorescence using Image J. Background fluorescence was determined for each image based on five randomly selected positions where no cells were visible, and the average value was subtracted from each fluorescence data measured for the given image. The X-axis indicates mean fluorescence (arbitrary units), while Y-axis denotes the number of cells. The dashed vertical line represents the median for each treatment.
